# Productivity responses of a widespread marine piscivore, *Gadus morhua*, to oceanic thermal extremes and trends

**DOI:** 10.1098/rspb.2009.1906

**Published:** 2010-02-10

**Authors:** Irene Mantzouni, Brian R. MacKenzie

**Affiliations:** 1 National Institute for Aquatic Resources, Technical University of Denmark (DTU-Aqua), Jægersborg Allé 1, Charlottenlund Castle, 2920 Charlottenlund, Denmark; 2 Department of Marine Ecology, University of Aarhus, c/o DTU-Aqua, Jægersborg Allé 1 Charlottenlund Castle, 2920 Charlottenlund, Denmark; 3 Center for Macroecology, Evolution and Climate, Department of Biology, University of Copenhagen, Universitetsparken 15, 2100 Copenhagen, Denmark

**Keywords:** cod, climate change, extreme events, meta-analysis, recruitment probability, temperature

## Abstract

Climate change will have major consequences for population dynamics and life histories of marine biota as it progresses in the twenty-first century. These impacts will differ in magnitude and direction for populations within individual marine species whose geographical ranges span large gradients in latitude and temperature. Here we use meta-analytical methods to investigate how recruitment (i.e. the number of new fish produced by spawners in a given year which subsequently grow and survive to become vulnerable to fishing gear) has reacted to temperature fluctuations, and in particular to extremes of temperature, in cod populations throughout the north Atlantic. Temperature has geographically explicit effects on cod recruitment. Impacts differ depending on whether populations are located in the upper (negative effects) or in the lower (positive effects) thermal range. The probabilities of successful year-classes in populations living in warm areas is on average 34 per cent higher in cold compared with warm seasons, whereas opposite patterns exist for populations living in cold areas. These results have implications for cod dynamics, distributions and phenologies under the influence of ocean warming, particularly related to not only changes in the mean temperature, but also its variability (e.g. frequency of exceptionally cold or warm seasons).

## 1. Introduction

‘Beef of the sea’, ‘British gold’ or ‘Newfoundland currency’ are some of the labels given to cod (*Gadus morhua*), a fish that has influenced world history and development for more than 1000 years (Kurlansky 1997). ‘Collapse’, however, has also characterized many north Atlantic cod stocks, owing to their pronounced decline for the last two decades (Christensen *et al*. 2003). High fishing pressure has been a major factor responsible for the present state of cod stocks but it is also recognized that climatic variability, by altering stock and ecosystem productivity, has also played an important role (Rothschild 2007; Lilly *et al*. 2008; Mieszkowska *et al*. 2009). Temperature is a key climatic component driving these fluctuations (Dutil & Brander 2003; Brander 2007), with immediate implications for cod recruitment (Pepin 1999; Planque & Frédou 1999).

The sensitivity of cod recruitment to temperature has been studied for various individual north Atlantic stocks and the identified mechanisms include direct and indirect impacts, such as changes in spawning time, reproductive capacity, growth rate of larvae and adults, recruitment success, distribution, prey abundance and plankton production (Sundby 2000; ICES 2008; Mieszkowska *et al*. 2009). Evidence for the geographical pattern of the effect has been provided by studies jointly considering a number of cod stocks (e.g. Planque & Frédou 1999). Similar patterns have also been found for temperature-dependent traits affecting recruitment, such as growth rate (Brander 1995), somatic condition (Rätz & Lloret 2003) and overall population productivity (Dutil & Brander 2003). Thus, global warming, which is having impacts on the phenology and distribution of hundreds of species (Parmesan 2006), also is raising concerns for the future sustainability of cod populations (Drinkwater 2005).

Another important aspect related to climate change is the increase in magnitude and/or frequency of extreme events (Easterling *et al*. 2000; IPCC 2007) and thus the higher probability of environmental conditions exceeding critical physiological and life-history thresholds (Jentsch *et al*. 2007). Extreme events, such as exceptionally cold or warm years, can impose profound impacts on the structure and functioning of ecosystems (Parmesan *et al*. 2000; Jentsch *et al*. 2007). Over time, these events and the biological responses will lead to changes in species distributions and could shift populations and ecosystems into new equilibria or regimes (Easterling *et al*. 2000; Parmesan *et al*. 2000). The ecological consequences of extreme events on marine biota have so far been relatively less thoroughly investigated compared with the number of studies investigating consequences of changes in trends and mean values (Easterling *et al*. 2000).

In the present study, our objective is to assess the biogeographic pattern of how cod recruitment has been influenced by spawning-season temperature, both trends and extreme events, in the past 20–40 years throughout the north Atlantic. We use two approaches to estimate two types of impacts. First, we suggest and implement a novel method in fisheries research to study effects induced by *thermally extreme* spawning seasons. In this context, we address two types of questions: (i) whether the probability of relatively stronger year-classes is enhanced by extremely cold or by warm spawning periods and (ii) whether and to what extent average recruitment survival differs between the coldest and warmest spawning seasons. Our second approach is based on standard correlational analyses of the long-term effects imposed by temperature *trends* on cod recruitment. This analysis considers, for the first time, to our knowledge, all 21 stocks and provides an update to current knowledge provided by earlier studies using subsets of available population data (Planque & Frédou 1999; Brander 2000). Most importantly, we use formally structured meta-analytic techniques (Cooper & Hedges 1994), rather than comparative approaches, to synthesize the effects across stocks and produce overall patterns that also accommodate the uncertainty in the stock-specific estimates.

## 2. Material and methods

### (a) Recruitment success indices

We are primarily interested in studying the response of cod recruitment (REC) to temperature variations owing to either the impact of thermal extreme events or trends. Because spawner stock biomass (SSB) might have linear and nonlinear effects on juvenile cod production rate (Cushing 1996; Myers & Barrowman 1996), we use natural log transformed recruitment survival (i.e. log (REC/SSB)) and also the residuals of a spawner-recruit model (Ricker 1954) as recruitment indices in addition to the raw recruitment data. Inference about the potential influence of temperature on these SSB standardized recruitment indices could be obscured in cases where temperature has an impact on both recruitment and SSB, and the effects are of similar strength and operate in the same direction. Hence, when the results obtained using recruitment survival and Ricker residuals were not significant, we also checked whether this result was owing to temperature effects on recruitment and SSB separately. Both variables were natural log transformed to stabilize variances. The SSB and REC data for the 21 major cod stocks in the north Atlantic were extracted from published stock assessment reports (see the electronic supplementary material for details).

### (b) Temperature

We focused on the effects of temperature (*T*) during the cod spawning season, given that the early life-history stages are most sensitive to abiotic environmental variations (Cushing 1996) and that recruitment success in many stocks and years is determined during the egg–larval–pelagic juvenile phase (Brander 2003). Thus, we used time series of spring (March–May) temperature, a period corresponding to the peak spawning time of cod for most stocks and years (ICES 2005). We assumed that the upper water layer between 0 and 100 m was representative of the pelagic environment experienced by cod pre-recruits (eggs, larvae and pelagic juveniles, corresponding approximately to ages 0 to approximately 3–4 months). Temperature data were compiled from large international and national oceanographic databases (see the electronic supplementary material for details).

### (c) Groups of stocks: cold and warm stocks

Temperature effects on cod recruitment have been shown to be stronger and opposite, at the lower and upper ends of the cod thermal distributional range (Planque & Frédou 1999; Brander 2000). Thus, we defined two groups of stocks, ‘warm’ and ‘cold’, including stock data within the upper or the lower temperature intervals, respectively. The intervals were defined based on the overall 25th and the 75th percentiles of temperature across the species range. The cold group includes stock-specific data corresponding to *T* < 4°C while the warm group includes observations made at *T* > 6.5°C. Five stocks (cod-gom, cod-4vsw, cod-4x, cod-coas and cod-kat; electronic supplementary material, table S1) do not have a sufficient (nine or more) number of observations in either temperature range and thus are not included in the groups.

### (d) Meta-analyses

Random-effects meta-analyses were used to study the effects of (i) thermally extreme events (i.e. exceptionally warm or cold springs) and (ii) temperature trends on recruitment success indices. Meta-analysis across cod stocks is possible through the encoding of the separate (stock-specific) responses on standardized, dimensionless metrics, the effect sizes, quantifying the degree and the direction of an effect, so that they can be subject to common statistical meta-analytic inference (Cooper & Hedges 1994). The effect sizes used are summarized in table 1 and further details for the methods are given in the electronic supplementary material.

**Table 1.** Interpretations for the metrics analysed with random effects meta-analysis (see the electronic supplementary material for further details on the estimation).

effect size	interpretation
risk ratio	null hypothesis: RR = 1: equal probabilities (risks) of successful events (recruitment survival above the stock specific mean or positive residuals) during extremely warm (exposed or hot group; *T* > *T*_75th%ile_) and cold (control or cool group; *T* < *T*_25th%ile_) seasons
	alternative hypothesis: RR > 1: higher probability (risk) of successful events during warmer seasons, and vice versa for RR < 1
Hedge's *g*	null hypothesis: average recruitment survival (or REC or SSB) does not differ between extremely cold (control or cool group; *T* < *T*_25th%ile_) and warm (exposed or hot group; *T* > *T*_75th%ile_) seasons
	alternative hypothesis: HG < 0: mean recruitment survival (or recruitment or SSB) is lower during relatively warm seasons, and vice versa for HG > 0
	the HG can be transformed into Cohen's *d* (CD) (Cohen 1988; Cooper & Hedges 1994: 239). The CD can be interpreted as (i) the per cent of non-overlap between the distributions of the observations during the cool and hot seasons, respectively, or (ii) as the percentile of the cool group distribution at which the mean of the hot group is standing
Fisher's *z*	FZ transform of the correlation coefficient (*r*_*i*_) between the stock time series or Ricker model residuals and temperature

#### (i) Thermally extreme events

Meta-analyses evaluating impacts of extreme events were based on the assumption that if temperature can influence recruitment, then the impacts will be mostly evident during the coldest and/or warmest spawning seasons at a given location. Warm seasons (termed as ‘hot’ or ‘exposed’) for a given stock are characterized as those when *T* exceeds the stock-specific 75th percentile (*T*_75%ile_), while cold seasons (termed as ‘cool’ or ‘control’) are defined as those with *T* below the stock-specific 25th percentile (*T*_25%ile_). The choice of the exact percentiles is meant to derive an empirical basis for categorizing years when local cod biology was subjected to particularly unusual thermal conditions, which deviated substantially from the average, to which the life history and physiology of local populations are most likely adapted. Another advantage of using percentiles is that the two groups have equal numbers of observations. Our purpose is to compare (i) the probability of strong year-classes and (ii) the average year-class strength between seasons of extremely low and high temperature and to identify the most favourable conditions. Thus, we used the risk ratio (RR) and Hedges' *g* (HG) effect-sizes and also the Wilcoxon signed-rank test (table 1; see also methods in the electronic supplementary material).

#### (ii) Thermal trends

Meta-analyses evaluating impacts of thermal trends were based on the correlation between temperature and recruitment survival. We used the Fisher's *z* (FZ) correlation coefficient as the effect size (table S1, electronic supplementary material). Special techniques and additional analyses were employed to address the possible influence of autocorrelation in the stocks time series, as explained in the electronic supplementary material.

## 3. Results

For each effect size, the meta-analyses were conducted separately for the warm (*n* = 7) and cold (*n* = 9) groups of stocks, using the SSB standardized measures of year-class strength (i.e. natural log of recruitment survival and the Ricker model residuals). Significant results were mainly obtained for the warm stocks, while in the cold group the results were close to significant only for FZ meta-analysis using the Ricker model residuals. As explained in §2, concurrent thermal effects on both recruitment and SSB can obscure the results for the SSB standardized indices. Thus, for the cold group, we also analysed the effect sizes estimated for the two variables separately. Overall, our results show that temperature extremes and trends induce negative effects on the recruitment dynamics of the warm stocks, and vice versa for the cold stocks. The meta-analyses were checked for robustness to the exclusion of any stock by performing sensitivity analyses, and the results were generally satisfactory. We also performed cumulative meta-analyses, by progressively adding stocks with increasing ‘coldness’ or ‘warmness’ (electronic supplementary material, table S1), to demonstrate how the effects become stronger when considering stocks closer to the extremes of cod thermal distribution.

### (a) Effects of thermally extreme events (HG, RR and Wilcoxon signed-rank test)

**Figure 1.**
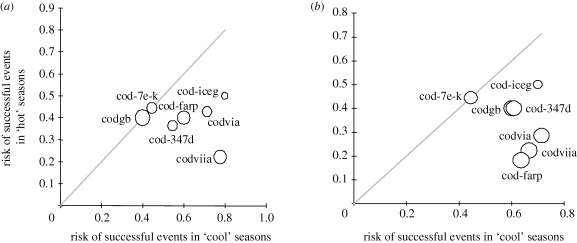
The proportion (risk) of the successful events (recruitment survival, log(REC/SSB)), above the stock-specific mean or positive residuals, during the hot (*T* > *T*_75th%ile_) seasons versus the corresponding risk during cool (*T* < *T*_25th%ile_) seasons of warm cod stocks: (*a*) survival (log(REC/SSB)) and (*b*) Ricker model residuals. The size of the points is proportional to the weight of the stock-specific RR estimates in the meta-analysis. The grey line through the origin is the 1 : 1 line corresponding to equal risks, i.e. no effect of cold or warm seasons on the year-class strength. For stock codes, see electronic supplementary material, table S1.

Extreme high temperatures (*T* > *T*_75th%ile_) during the spawning season were shown to induce negative effects on recruitment dynamics of the warm stocks. Specifically, employing the RR (table 1) meta-analyses, it was found that the probabilities of low (i.e. below the stock-specific mean) recruitment survival (log(REC/SSB)) and negative Ricker residuals were significantly higher during the relatively warmer seasons, by 36 per cent (log(RR) = −0.44) and 41 per cent (log(RR) = −0.53), respectively, compared with the seasons with lower *T* (electronic supplementary material, figure S2 and table S2). Similarly, when plotting the probabilities during warmer seasons versus the corresponding probabilities during the colder seasons, most of the points fall below the no-effect line, indicating that the probability of ‘successful’ events is higher during colder spawning seasons (figure 1).

**Figure 2.**
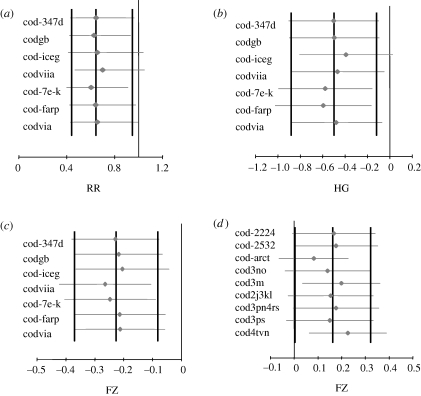
Exclusion sensitivity plots for the meta-analyses of temperature effects on recruitment survival of the warm cod stocks using (*a*) RR, (*b*) HG and on the Ricker model residuals using FZ for the (*c*) warm and (*d*) cold stocks. Exclusion sensitivity analyses are performed by excluding one stock at a time from the analysis (the one correspondingly denoted on the left column) and repeating the meta-analysis with all the remaining ones. The results with confidence intervals (CIs) (95% for (*a*–*c*) and 90% for (*d*)) are plotted with grey symbols and grey horizontal lines. The vertical black lines correspond to the final meta-analytic result estimated based on all the stocks of each group and are plotted for comparison. For stock codes, see electronic supplementary material, table S1.

The exclusion sensitivity plot of the Ricker residuals meta-analysis revealed that the result is in general robust and mostly sensitive to the Icelandic and Irish Sea cod (figure 2*a*). The result, however, remains significant at *p* < 0.1 even if either of the stocks is excluded from the analysis. The cumulative of the Ricker residuals meta-analysis showed that the overall RR becomes significantly lower than 1 after including stocks with mean spring *T* > 7.5°C in the meta-analysis (electronic supplementary material, figure S3*a*). The sensitivity and cumulative analyses for warm stocks recruitment survival were similar. The results remained significant (*p* < 0.1) when certain observations were excluded in order to eliminate autocorrelation (see methods in the electronic supplementary material) from those stocks (cod on Faroe Plateau and Icelandic cod) with substantial autocorrelation in the recruitment survival and Ricker residuals (electronic supplementary material, table S1).

**Figure 3.**
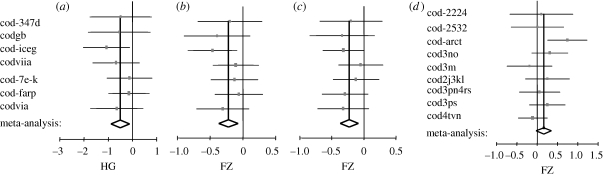
Stock-specific (squares) and the overall (diamond) effect sizes with CIs estimated with random effects meta-analysis of (*a*) HG of the warm stocks recruitment survival (log(REC/SSB)), (*b*) FZ of warm stocks (log(REC/SSB)), (*c*) FZ of warm stocks Ricker model residuals and (*d*) FZ of cold stocks Ricker model residuals. In plots (*a*–*c*), the 95% CIs are plotted while in (*d*) the 90% CIs are given. The vertical black lines correspond to the mean overall effect size and is plotted for comparison with the individual estimates. The width of the diamonds represent the CIs of the overall effect size. The size of the squares is proportional to the weight of the individual effect sizes in the meta-analysis. For stock codes, see electronic supplementary material, table S1.

The HG (table 1) meta-analysis revealed that the mean recruitment survival of warm stocks was significantly higher during relatively colder (cool) compared with warmer (hot) spawning seasons, and the difference is about 50 per cent of the observations standard deviation pooled between the colder and warmer seasons (figure 3*a* and table 2; electronic supplementary material, table S2). The exclusion sensitivity plot showed that, similar to the RR meta-analysis, the result was robust in general, since the results would become insignificant at *p* = 0.05 (but still remain significant at *p* = 0.1) only if Icelandic cod, which is among the stocks with higher sample sizes and weights in the analysis, was excluded (figure 2*b*). The cumulative meta-analysis showed that the pattern becomes evident after including stocks with mean *T* > 7.1°C (electronic supplementary material, figure S3*b*). The corresponding overall Cohen's *d* (CD; table 1) is −0.53, indicating that the mean survival during higher *T* conditions is approximately at the 30th percentile of the observations at cool spawning seasons.

**Table 2.** Random-effects meta-analysis results for the RR, HG and FZ per group of stocks. (The sample size refers to the total number of observations (equal to the total number of extremely low or high temperature observations across cold and warm stocks for RR and HG), summed across the stocks included in the analysis. Note that for the RR the results refer to natural log-transformed RR. See table 1 for the interpretation of the effect sizes.)

effect size	time series (sample size)	meta-analysis outcome	standard error	*p*-value
**warm stocks (*T* > 6.5°C)**				
RR	log(REC/SSB) (112)	−0.44	0.20	0.03
RR	residuals (112)	−0.53	0.21	0.01
HG	log(REC/SSB) (112)	−0.50	0.19	0.01
FZ	log(REC/SSB) (219)	−0.22	0.08	<0.01
FZ	residuals (219)	−0.23	0.07	<0.01
**cold stocks (*T* < 4°C)**				
RR	log(REC) (120)	0.56	0.22	<0.01
RR	log(SSB) (120)	0.67	0.25	<0.01
HG	log(REC) (120)	0.69	0.21	<0.01
HG	log(SSB) (120)	0.77	0.23	<0.01
FZ	log(REC) (227)	0.27	0.11	0.01
FZ	log(SSB) (227)	0.23	0.11	0.04
FZ	residuals (227)	0.16	0.10	0.10

The one-sided Wilcoxon signed-rank test (see methods in the electronic supplementary material) showed that, for the warm stocks, the median of the distribution of the log-transformed ratios between average recruitment survival during colder and warmer seasons is significantly negative (−0.058, *p* < 0.01). This result agrees with the HG meta-analysis, revealing that the average log(REC/SSB) is significantly lower at higher spring *T* conditions.

For the cold stocks, both RR and HG meta-analyses showed that extreme high temperatures impose positive effects on both recruitment and SSB (table 2), and thus no effect was detected for the SSB standardized indices of recruitment survival.

### (b) Effects of temperature trends (FZ)

The across warm stocks correlation of both recruitment survival and Ricker residuals with spring temperature, corrected for autocorrelation at lag 1 (see the electronic supplementary material), was found significantly negative (figure 3*b*,*c* and table 2). The results were stronger using the latter index and are thus mainly presented. The exclusion sensitivity plot (figure 2*c*) shows that the result is robust to the exclusion of any stock. The pattern emerges after the stocks with average spring *T* > 7.1°C are included in the analysis (electronic supplementary material, figure S3*c*).

As in the previous meta-analyses for the cold stocks group, significant results (*p* < 0.05) were obtained for both the log-transformed recruitment and SSB (table 2), which appear to be positively correlated with the spring temperature. In addition, however, it was shown that there is a close to significant (*p* = 0.10) positive correlation between the Ricker residuals and spring *T* (table 2 and figure 3*d*). When either cod-3m or cod-4tvn, displaying negative FZ, is excluded from the analysis, the result becomes significant at *p* < 0.5 (figure 2*d*). The pattern becomes evident after stocks with average *T* < 4°C are included in the analysis (electronic supplementary material, figure S3*d*).

## 4. Discussion

The present study, to our knowledge, is the first attempt to meta-analyse nearly all 21 north Atlantic cod stocks, in order to identify statistically the effect of temperature, especially the impact of extreme thermal conditions on cod recruitment. This investigation is also one of the few climate-change-related studies addressing productivity and abundance, rather than distributional changes, throughout the range of a marine fish species. Using meta-analytic methods, we showed that temperature during the spawning season (spring) is an important factor affecting cod recruitment, reproductive success and spawner abundance, and we provided new evidence on the geographical patterns of these effects. Effects were prominent when considering either (i) the correlation between the temperature and population trends or (ii) the influence imposed by single events of exceptionally warm or cold seasons.

The results show that the direction of the effects imposed by increased temperature, both trends and extreme events, is opposite in the upper (negative) and the lower (positive) thermal limits. In particular, high thermal events are associated with lower recruitment survival (and negative Ricker model residuals) for the warm stocks (*T* > 6.5°C; *n* = 7), compared with when spawning season temperature is relatively low. The pattern is opposite for the cold stocks (*T* < 4°C; *n* = 9), with both recruitment and SSB being positively affected in warmer seasons. Correlation between cod biomass and temperature has also been found in a previous meta-analytic study (Worm & Myers 2003) and can be driven by the temperature effects on survival, recruitment and/or growth rates (Brander 1995; Worm & Myers 2003). Owing to the impacts on SSB in the cold stocks, no significant results were obtained within this group for recruitment survival or for the Ricker model residuals. However, there were some indications of positive correlation between the Ricker residuals and the spring temperature.

The main motives for investigating the sensitivity of cod recruitment to extreme temperature conditions were (i) the frequency of warm and cold seasons and/or years in some areas of the north Atlantic has changed in the last 120–140 years (MacKenzie & Schiedek 2007), while (ii) relatively few studies investigate how extreme events affect marine ecological responses in nature. Extreme events, via impacts on life histories and physiologies, may influence key prey or predators of a species and thereby relax or strengthen food-web controls on species distributions. In addition, changes in extreme conditions may be more important than changes in means for the initial establishment (or maintenance) of populations in new (or existing) areas (e.g. abiotic conditions reaching threshold levels to which all life-history stages of a species can tolerate). According to the ‘species range hypothesis’ (Huffaker & Messenger 1964), impacts of environmental fluctuations are most prominent at the edges of a species distribution, where the conditions are more likely to reach unfavourable values and thus, populations can be more sensitive to density-independent factors.

Therefore, part of our analyses focused on the effects of extreme temperature both across and within stocks. We compared cod dynamics for distinct cold and warm groups of stocks during (relatively) colder and warmer spawning seasons. The implemented methods, based on effect sizes quantifying and contrasting the magnitude and direction of extreme thermal events in natural populations, could be useful for the investigation of such effects in other single populations and/or, when combined with meta-analytic approaches, across a species range.

Our results illustrating the consequences of extreme temperature on recruitment are consistent with recent studies investigating thermal habitats experienced by wild cod, based on data storage tagging experiments in the north-east Atlantic (Neat & Righton 2007; D. Righton 2009, personal communication). These studies showed that the thermal range cod experienced throughout the year, within and among ecosystems, was large, thereby demonstrating that this species has a physiological tolerance to broad temperature conditions. However, the thermal range varied at seasonal scales, and was much narrower in the months prior to and during spawning, with a peak at 7°C. These observations suggest that reproduction is more sensitive to temperature than processes affecting cod physiology and ecology at other times of year (Pörtner & Farrell 2008). Our decision to use temperatures during the spawning period as a possible indicator of thermal conditions affecting cod recruitment is therefore consistent with the direct individually based observations of thermal habitats actually occupied by wild cod in north-east Atlantic ecosystems.

It is notable that the peak temperature observed by D. Righton 2009 (personal communication) during the cod spawning period (7°C) was close to the 75th percentile of spawning season temperature observed across the species range (6.5°C), using independent temperature datasets, and which we *a priori* used to designate the warm group of cod stocks. We believe therefore that the two studies (Righton *et al*. and our present one) both indicate that temperatures during the spawning period exceeding 6.5–7°C may be detrimental for reproductive success. Similarly, we have also observed in a separate study using hierarchical Bayesian analyses of cod population dynamics that the maximum reproductive rate of cod in these same 21 populations also declines when spring temperatures exceed 5–6°C (Mantzouni *et al*. in press). Moreover, the temperature interval (6–7°C) at which the macro-scale distribution of relative frequencies of a well-studied cod allele (*Pan* I) changes (Case *et al*. 2005) is similar to these ranges. Such warm temperatures may be detrimental either for the reproductive physiology of adult cod (e.g. gonadal development) or for the survival of eggs and larvae. Alternatively, cod life history may have evolved to spawn within a narrower and relatively low range of temperatures because other ecosystem and food-web processes that subsequently affect the survival of the eggs and larvae (e.g. production rates of predators and prey of the eggs and larvae) may be closely correlated with this range of temperature. Although temperatures in the different studies are not exactly from the same times of year or depths, they were all measured at the main spawning locations and times. Thus, we believe that there exists sufficient comparability among the datasets to suggest that there are now several independent pieces of evidence (genetic, behaviour/migratory, population dynamics) advancing our knowledge on the processes by which cod populations are affected by spawning season temperature variability across an extensive spatio-temporal scale. Resolving the mechanisms of exactly which processes are influenced by temperature, and their potential spatial differences throughout the north Atlantic, will require new process-specific and modelling studies.

The predicted amplification of the extreme high thermal events and the reduction in the comparatively colder events (Easterling *et al*. 2000; IPCC 2007; Jentsch *et al*. 2007) is expected, based on our results, to induce opposite effects on cod dynamics, depending on their location; negative effects will be imposed on recruitment productivity of stocks inhabiting relatively warmer waters, while recruitment in colder waters will probably increase, probably through positive effects on cod spawner biomass. Impacts will also include a range of responses at various levels of biological organization, including behavioural responses to occupy suitable habitats (Neat & Righton 2007; Dulvy *et al*. 2008; D. Righton 2009, personal communication), physiological (e.g. growth) responses (Brander 2000) and potentially evolutionary responses via changes in genotypes with different tolerances to thermal stresses (Case *et al*. 2005; Nielsen *et al*. 2007). High fishing pressure, in addition, can amplify the sensitivity of populations to climate-induced stress, by altering their abundances or demographic (Anderson *et al*. 2008) and/or geographical structures and reducing the biodiversity of ecosystems (Perry *et al*. 2005; Planque *et al*. 2010). Thus, quantifying environmental effects on fish productivity, and investigating how fisheries reference parameters (e.g. recovery rates, maximum biomasses in given ecosystems) depend on climatic variables, could provide useful insights for fisheries management.

Nevertheless, even at favourable thermal conditions and reduced fishing, a variety of mechanisms and factors can prevent collapsed populations from recovering (Caddy & Agnew 2004), including changes in predator–prey relationships (Swain & Chouinard 2008), regime shifts in ecosystem structure and function (Frank *et al*. 2005) and Allee effects (Frank & Brickman 2000; De Roos & Persson 2002) associated with population and predator–prey size structure relationships. These mechanisms can stabilize populations at low biomass. Thus, ecosystem-level research is needed to understand the relative importance of exploitation, community mechanisms and environment for species present and future state.

The presented multi-population level responses to extreme and mean temperature trends and the patterns in the spatial dependence of recruitment dynamics on temperature are difficult to resolve by single-stock analyses or by pooling all stock-recruitment data across stocks and treating the individual populations as one large undifferentiated population. These difficulties were mostly overcome by the use of meta-analyses, since patterns that are weak or not significant at local scales of single stocks, but which nevertheless are repeated in different geographical areas, or vary systematically across space, may generate significant and ecologically meaningful patterns at larger scales of organization (e.g. Frank *et al*. 2007). Joint analyses have also illuminated the patterns of North Atlantic Oscillation on cod recruitment (Brander & Mohn 2004; Brander 2005; Stige *et al*. 2006). Thus, meta-analysis is also an effective approach for the study of regional and global environmental change (Young *et al*. 2006; Rudel 2008). In particular, the identification of temperature effects on residuals from the Ricker model suggests that new insights could be gained by the development of stock-recruit models in a meta-analytic framework to investigate how Ricker model parameters (e.g. maximum reproductive rate, carry capacity) depend on environmental variables (Mantzouni *et al*. in press).

Climate change has been shown to have different levels of impact on different species, depending on their life-history traits and physiological tolerances (Parmesan & Yohe 2003). Thus, it is possible and indeed likely that the impacts on a species, such as cod, will be partly mediated through the food web, by effects on prey, predators and pathogens (Worm & Myers 2003; Parmesan 2006), depending on their sensitivities relative to those of cod. In this sense, new meta-analyses of how species interactions in food webs are affected by environmental variables, including temperature, salinity, primary productivity and higher order system variables such as biodiversity (e.g. Frank *et al*. 2007) could provide new insights into the stability and resilience of both populations and ecosystems to perturbations, including those due to climate change. Such analyses would contribute to macro-ecological scales of understanding how marine ecosystems are structured and function and will be increasingly needed for ecosystem management as climate change progresses.
